# Atrioventricular Block Caused by Carteolol Eye Drops Administered to Unilateral Eye or Bilateral Eyes: A Case Report

**DOI:** 10.7759/cureus.90018

**Published:** 2025-08-13

**Authors:** Keiji Yoshioka, Tatsuya Kawasaki

**Affiliations:** 1 Diabetes and Endocrinology, Yoshioka Diabetes Clinic, Osaka, JPN; 2 Cardiology, Matsushita Memorial Hospital, Moriguchi, JPN

**Keywords:** atrioventricular blocks, beta-blocker, carteolol, diabetes, eye drops

## Abstract

Ophthalmic beta-blocker carteolol can rarely cause cardiovascular effects such as bradycardia, heart block, and hypotension. We report a case of carteolol eye drops induced atrioventricular (AV) block. A 76-year-old man with type 2 diabetes developed exercise-induced second-degree AV block, which improved after discontinuing carteolol eye drops in the unilateral eye. Six months later, third-degree AV block recurred due to inadvertent reuse of carteolol eye drops in bilateral eyes despite its prohibition. A seven-day patch electrocardiogram (ECG) monitor showed AV conduction returned to a 1:1 ratio after discontinuation of eye drops. The pacemaker implantation was deferred, and he is currently under close follow-up. Depending on the amount administered to a unilateral eye or bilateral eyes, topical carteolol eye drops can cause second- or third-degree AV block.

## Introduction

It is known that topical beta-blockers such as timolol and carteolol can cause systemic cardiovascular events, including bradycardia, heart block, and hypotension [1], and elderly patients [2], especially with diabetes [3] or chronic kidney disease (CKD) [4], may be more susceptible to atrioventricular (AV) block. Topical beta-blocker-induced AV block has been reported to occur less frequently with carteolol [5] than with timolol eye drops [6-8]. This is due to the intrinsic sympathomimetic activity (ISA) of carteolol itself, which refers to the property of some beta-blockers to partially stimulate beta-adrenergic receptors while also blocking them [9]. Polypharmacy is common in older people, especially with multiple comorbidities, and is associated with an increased risk of systemic adverse events due to drug interactions. Physicians should always be aware of all prescribed medications, but non-oral ophthalmic medications may be overlooked when physicians are not in close communication with each other. We report a case of an elderly man with type 2 diabetes and CKD who was initially diagnosed with carteolol eye drop-induced second-degree AV block in the unilateral eye but developed third-degree AV block due to the inadvertent reuse of carteolol eye drops in bilateral eyes, despite reporting the initial event to his ophthalmologist.

## Case presentation

A 76-year-old man with a 36-year history of type 2 diabetes presented with palpitations and shortness of breath on exertion that he had not previously experienced. He had no history of coronary artery disease or other structural heart disease. The patient was receiving empagliflozin (10 mg/day), degludec/liraglutide (~30 doses/day), and ezetimibe (10 mg)/atorvastatin (10 mg) for the treatment of diabetes and dyslipidemia in the outpatient diabetes clinic. He was not on any other oral medications.

On physical examination, his blood pressure was 138/90 mmHg, and his heart rate was 69 bpm. Heart sounds were normal, and there was no murmur. Respiratory sounds were unremarkable. At the initial consultation two years ago, orthostatic hypotension was noted with a decrease in systolic blood pressure of -22 mmHg on standing. The laboratory data showed decreased estimated glomerular filtration rate (eGFR) at 41.0 ml/min/1.73 m² with marked proteinuria. The glycated hemoglobin A1c level was 8.9%, and liver function and electrolyte levels were within normal limits.

**Figure 1:**
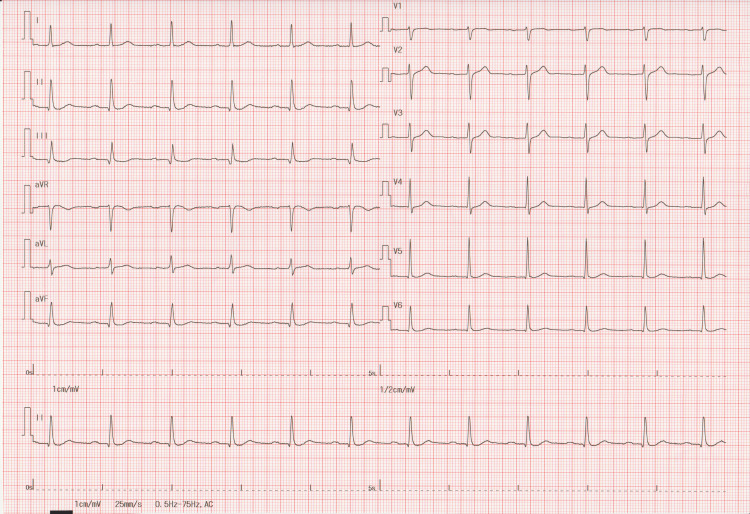
Master’s double two-step test during the first episode An electrocardiogram before exercise shows a normal sinus rhythm without ST-T segment changes.

**Figure 2:**
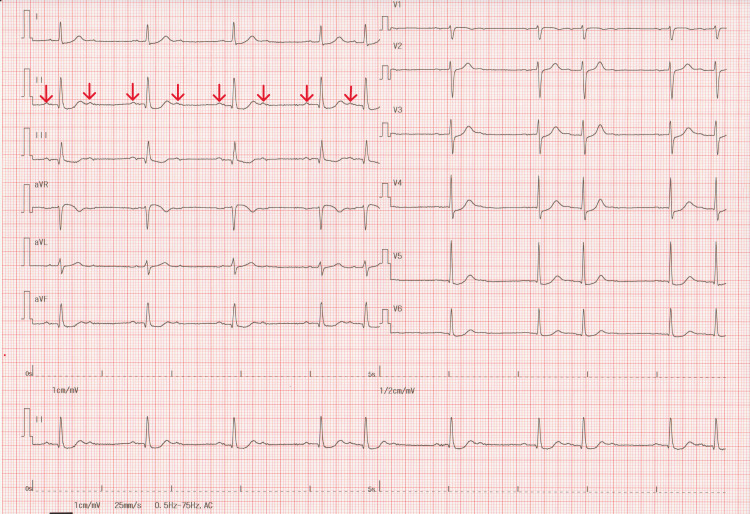
Master’s double two-step test during the first episode An electrocardiogram immediately after exercise shows a second-degree AV block with 2:1 conduction (red arrows), accompanied by significant ST depression in leads I, II, aVF, V5, and V6.

**Figure 3:**
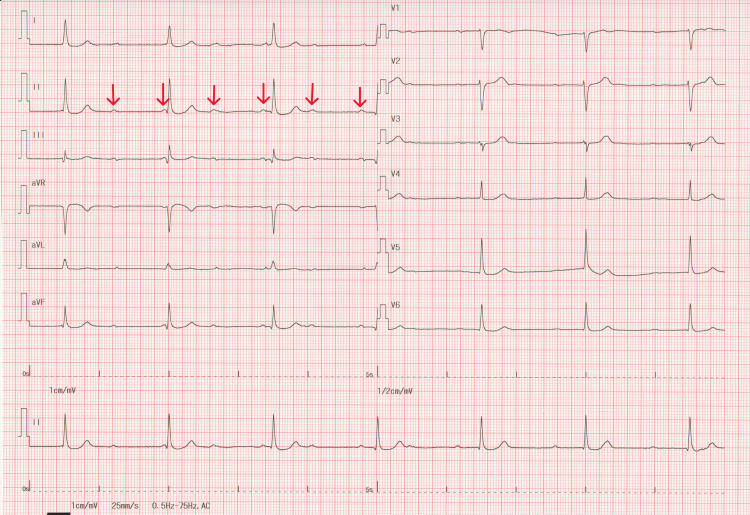
Electrocardiogram during the second episode An electrocardiogram shows a third-degree AV block (red arrows) with a ventricular rate of 40 bpm.

A 12-lead electrocardiogram (ECG) at rest showed a sinus rhythm with a 70 bpm heart rate and no ST-T changes (Figure 1) that were similar to the ECG performed two years earlier, with a coefficient of variation of heart rate (coefficient of variation of R-R intervals) of 1.42%. However, immediately after the Master’s double two-step test, a second-degree AV block with 2:1 conduction appeared with significant ST-depression in leads I, Ⅱ, aVF, V5, and V6 (Figure 2). After three minutes of recovery, AV conduction returned to 1:1, and the ST-T changes normalized. The patient was then referred to a cardiologist for further evaluation. Transthoracic echocardiography revealed a normal ventricular function (left ventricular ejection fraction of 59%) with no LV dilatation or valvular disease. Exercise thallium-201 myocardial scintigraphy did not reveal any significant myocardial perfusion defects. No further coronary angiography was performed. After re-reviewing all prescribed drugs to see whether they could cause AV block, it was determined that topical carteolol eye drops (2% concentration solution) prescribed by his ophthalmologist for glaucoma were a possible cause of exercise-induced second-degree AV block. The patient had instilled one drop into the unilateral eye once daily, but it was unclear whether the nasolacrimal occlusion technique was strictly adhered to. The topical carteolol was discontinued, and thereafter, AV blocks were no longer evident on 24-hour Holter monitoring. We reported this topical carteolol-induced heart block adverse event to his ophthalmologist. The patient had no symptoms for the next six months after discontinuing the carteolol eye drops, but the symptoms recurred. The ECG confirmed a third-degree AV block (Figure 3). To our surprise, he had inadvertently reused the carteolol eye drops in bilateral eyes, despite reporting the initial event to his ophthalmologist. One week after discontinuation of the carteolol eye drops, an asymptomatic third-degree atrioventricular (AV) block persisted, and patch ECG monitoring was immediately initiated for seven days, which showed restoration of AV conduction returned to a 1:1 ratio after five days (i.e., 12 days after discontinuation). Pacemaker implantation was deferred, and he is currently under close follow-up.

## Discussion

Ophthalmic beta-blocker eye drops are widely used for the treatment of glaucoma or ocular hypertension. Approximately 80% of the drug can be absorbed into systemic circulation through the nasolacrimal ducts or conjunctival vessels without hepatic first-pass metabolism [10] and has been reported to reduce pulse rate by 7.61 bpm [11], although systemic concentration is low in comparison to that achieved with oral beta-blockers. A previous analysis evaluating systemic adverse events of beta-blockers used for glaucoma in 8,793 case reports showed bradycardia and complete AV block to occur in 1.88% and 0.43%, respectively [12]. It has been reported that the incidence of AV block is lower with carteolol [5] than with other eye drops [6-8] due to the intrinsic sympathomimetic activity (ISA) of carteolol itself [9].

The patient presented with poor glycemic control, chronic kidney disease (CKD) with a reduced eGFR of 41.0 ml/min/1.73 m², and macroproteinuria. A recent Danish nationwide registry study of 31,301 patients reported that the risk of third-degree AV block increased stepwise with decreasing eGFR, and CKD was associated with a higher incidence of third-degree AV block (HR: 1.83; CI: 1.73-1.93) independent of comorbidities and potential AV node blocking agents [4]. Carteolol is primarily excreted via the kidney [10]; decreased excretion may result in accumulation in the circulation, slowing myocardial conduction and predisposing to AV block, which may be one of the triggers in this case. Carteolol is metabolized through the CYP2D6 enzyme, and its beta-blocking efficacy is enhanced in elderly patients [10] or when used with CYP2D6 inhibitors such as calcium channel blockers; therefore, in many cases of heart block and cardiac events induced by topical beta-blockers, a calcium channel blocker such as verapamil [5] or benidipine hydrochloride [13] has been used in combination with topical beta-blockers. However, no such medication was administered in this case.

The first episode presented with exercise-induced second-degree AV block. It has been reported that pre-existing cardiac pathologies, such as myocardial ischemia or coronary artery vasospasm, can manifest as exercise-induced AV block [14,15]. The patient’s echocardiogram revealed normal left ventricular function, and exercise-induced thallium-201 myocardial scintigraphy showed no significant myocardial ischemia. In the first episode, transient exercise-induced second-degree AV block with 2:1 conduction occurred during topical carteolol eye drop treatment but resolved quickly after discontinuing the drug, indicating an iatrogenic cause.

There have been some reports that diabetes itself is an independent risk factor for third-degree AV block compared to controls [16]. Cardiovascular autonomic neuropathy (CAN), known as a diabetic microvascular complication, is a risk factor for silent myocardial ischemia, myocardial dysfunction, cardiac arrhythmias, and sudden death [17]. CAN prevalence increases with age, duration of diabetes, and poor glycemic control, which was also the case in this patient. Although serial heart rate variability metrics were not evaluated, the patient presented with orthostatic hypotension and reduced heart rate variability, suggesting the possibility of CAN. In the first episode, no cardiac conduction disturbances or cardiac events occurred before administration or after discontinuation of topical carteolol eye drops, so topical carteolol eye drops were determined to be the cause of second-degree AV block. However, six months after the initial attack, third-degree AV block caused by inadvertent reuse of carteolol eye drops in bilateral eyes recurred and persisted for several days even after carteolol eye drops were discontinued, so we cannot completely exclude the possibility that CAN may have contributed to prolonging recovery of the third-degree AV block during the second episode.

Various ophthalmic beta-blockers differ with regard to the presence or absence of ISA, lipid solubility, and renal excretion. Therefore, the incidence of ophthalmic beta-blocker-induced AV block may depend on these factors. In this case, despite carteolol ISA, older age, decreased eGFR (CKD), and possible CAN were possible risk factors for AV block. The onset of symptoms and AV block occurred after carteolol use and resolved after discontinuation of the drug; that confirms a causal relationship between the drug and AV block. On the other hand, monitoring serum carteolol concentrations and the relationship between presumed systemic exposure and AV block may be important, but there is a lack of relevant literature on this point in cases of eye drop-induced AV block, which limits the review of this case. Finally, Özcan KS, et al. [6] reported that the patients who presented with topical beta-blocker-induced AV block required high-rate pacemaker implantation during follow-up, so close follow-up is still required in this case.

## Conclusions

We experienced a case of second- or third-degree AV block caused by carteolol eye drops, depending on the dose administered to unilateral eye or bilateral eyes, in an elderly diabetic patient who had multiple comorbidities and an increased risk of susceptibility to drug-induced side effects.

This case highlights the importance of being aware of all prescribed medications that may cause AV block and the need for close communication between physicians.
